# Vertical Stratification of Sediment Microbial Communities Along Geochemical Gradients of a Subterranean Estuary Located at the Gloucester Beach of Virginia, United States

**DOI:** 10.3389/fmicb.2018.03343

**Published:** 2019-01-11

**Authors:** Yiguo Hong, Jiapeng Wu, Stephanie Wilson, Bongkeun Song

**Affiliations:** ^1^College of Environmental Science and Engineering, Guangzhou University, Guangzhou, China; ^2^Department of Biological Sciences, College of William & Mary, Virginia Institute of Marine Science, Gloucester Point, VA, United States

**Keywords:** microbial community, vertical stratification, spatial distribution, sediment core, subterranean estuary

## Abstract

Subterranean estuaries (STEs) have been recognized as important ecosystems for the exchange of materials between the land and sea, but the microbial players of biogeochemical processes have not been well examined. In this study, we investigated the bacterial and archaeal communities within 10 cm depth intervals of a permeable sediment core (100 cm in length) collected from a STE located at Gloucester Point (GP-STE), VA, United States. High throughput sequencing of 16S rRNA genes and subsequent bioinformatics analyses were conducted to examine the composition, diversity, and potential functions of the sediment communities. The community composition varied significantly from the surface to a depth of 100 cm with up to 13,000 operational taxonomic units (OTUs) based on 97% sequence identities. More than 95% of the sequences consisted of bacterial OTUs, while the relative abundances of archaea, dominated by Crenarchaea, gradually increased with sediment core depth. Along the redox gradients of GP-STE, differential distribution of ammonia- and methane-oxidizing, denitrifying, and sulfate reducing bacteria was observed as well as methanogenic archaea based on predicted microbial functions. The aerobic-anaerobic transition zone (AATZ) had the highest diversity and abundance of microorganisms, matching with the predicted functional diversity. This indicates the AATZ as a hotspot of biogeochemical processes of STEs. The physical and geochemical gradients in different depths have attributed to vertical stratification of microbial community composition and function in the GP-STE.

## Introduction

At the land-sea margin, coastal permeable sediments form the interface between the freshwaters of the coastal unconfined aquifers and seawater-derived saline pore water. Sandy permeable sediments cover over 70% of continental shelves (Gao et al., 2010). Sand beaches have been thought as geochemical deserts that could not support biogeochemical processes due to limited organic matter (Boudreau et al., 2001). However, recent studies have demonstrated that the permeable sediments are highly active in biogeochemical reactions due to special physiochemical properties such as steep redox, salinity and oxygen gradients, longer residence times, and stronger particle–water interactions (Moore, 1999; Santos et al., 2008; Gonneea and Charette, 2014; O’Connor et al., 2015; Seidel et al., 2015; Reckhardt et al., 2017). This mixing zone, home to a variety of important biogeochemical reactions, is referred to as a subterranean estuary (STE) (Moore, 1999). The biogeochemical processes occurring in STEs may regulate the fluxes of nutrients, organic matter, and metals within a submarine groundwater discharge (SGD) to coastal water (Charette et al., 2005; Charette and Sholkovitz, 2006; Santos et al., 2008; Santoro, 2010; Weinstein et al., 2011; Avery et al., 2012; Kim et al., 2012, 2013; Gonneea and Charette, 2014). STEs have been impacted by increased nutrient input from both fresh water and sea water, and changes of physicochemical conditions due to anthropogenic activities (Talbot et al., 2003; Slomp and Van Cappellen, 2004; Moore et al., 2008).

Biogeochemical processes occurring in STEs are mediated by biotic and abiotic reactions that include desorption of ions from adsorbed sites due to increases in ionic strength (Charette and Sholkovitz, 2002), dissolution and precipitation of carbonates (Liu et al., 2017), remineralization of organic matter leading to carbon, nutrient (Roy et al., 2013), and metal release (Seidel et al., 2015; O’Connor et al., 2018), oxidation-reduction reactions that produce and consume metal oxides (Charette et al., 2005; O’Connor et al., 2018), and the transformation of nutrients such as nitrogen and phosphate (Santos et al., 2008; Gao et al., 2012; Gonneea and Charette, 2014; Couturier et al., 2017). In contrast to surface estuaries, little is known about the STE microbial communities responsible for biotic reactions. Although it is now widely accepted that microbial communities are the foundation of biogeochemical cycling in the sandy permeable sediments, the possible contribution of microorganisms to the complex processes occurring in freshwater–seawater mixing zones has often been conjectured (Moore, 1999; Charette and Sholkovitz, 2002; Santos et al., 2008; Couturier et al., 2017).

The Gloucester Point STE (GP-STE), located in the Lower York River Estuary of Virginia, United States, contains fine grained and permeable sediments. O’Connor et al. (2015) reported the impacts of redox gradients on the speciation and mobility of redox-sensitive elements (RSEs). The redox gradients at different depths of the GP-STE can significantly affect microbial biogeochemical reactions including aerobic respiration, nitrification, denitrification, metal oxide reduction, methane oxidation and sulfate reduction. However, no study has reported the vertical distribution of microbial communities and their relationship with physical and geochemical characteristics in the GP-STE. We hypothesized that physicochemical conditions would significantly influence the structure and function of microbial communities, resulting in the GP-STE as a hotspot of biogeochemical reactions attenuating nutrients in SGD. The objectives of this study were to (1) examine the vertical distribution of microbial communities in permeable sediments, (2) access potential function of microbial communities based on metabolic inference, and (3) identify the geochemical controls on the microbial communities of the GP-STE. High-throughput sequencing of 16S rRNA genes was used to examine microbial community composition while the community function was inferred by a bioinformatic program, Phylogenetic Investigation of Communities by Reconstruction of Unobserved States (PICRUSt)^1^. Furthermore, statistical analyses were performed to identify the physical and geochemical features influencing microbial community structures and their potential functions.

## Materials and Methods

### Site Description, Samples Collection, and Physicochemical Parameters Analysis

The study site is located at the mouth of the York River Estuary at Gloucester Point (37.248884 N, 76.505324 W), VA, within the Chesapeake Bay. The details of the site description were previously reported by Beck et al. (2016). SGD rates ranged from 3.9 to 8.9 cm day^-1^ depending on discharging locations (Luek and Beck, 2014; Beck et al., 2016). The GP-STE contains fine grained and permeable sediments.

**Figure 1:**
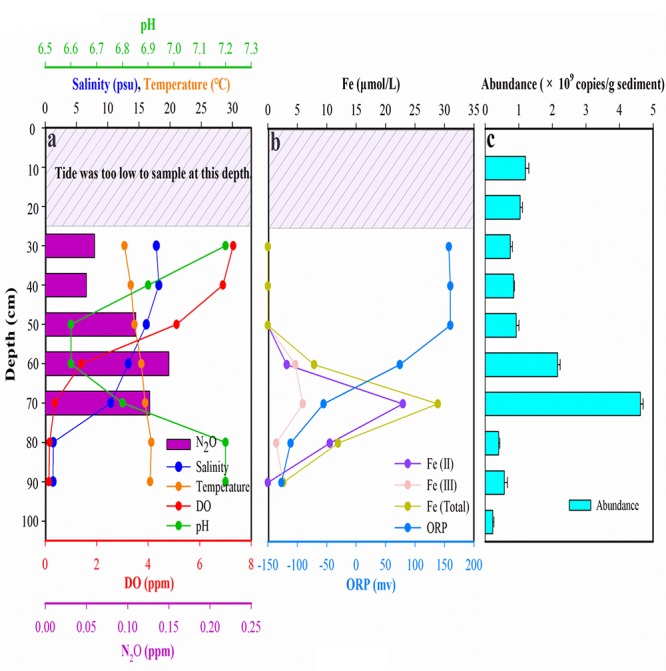
Environmental parameters and microbial abundance measured at different sampling depths of the sediment cores from Gloucester beach. **(a)** The salinity, temperature, DO, pH, and N_2_O concentration of groundwater. **(b)** The concentration of dissolved Fe [Fe(II), Fe(III), and Fe (Total)] and ORP values in groundwater. **(c)** Microbial abundance detected by 16S rRNA gene in sediment core. Tide was too low to sample the ground water in surface sediment (0–25 cm), so the groundwater samples of surface sediments were missing.

A sediment core, about 1.0 m in length, was collected using a vibrocorer in October of 2015. Sediments in a single core were sub-sectioned at an about 10 cm interval, for a total of 10 subsamples (Figure 1, Table 1, and Supplementary Figure S1) were obtained and stored at -80°C until further analysis.

**Table 1:** Comparison of diversity and coverage estimates of the sediment communities of Gloucester beach based on 3% dissimilarity level of 16S rRNA gene sequences for the sediment permeable intertidal samples collected from Gloucester Point.

Samples	High-quality reads	OTU^a^	Chao1^a^	ACE^a^	Shannon^a^	Evenness^b^	Coverage^a^
GP-1	28,178	5,744	10,577	9,881	7.10	0.82	0.89
GP-2	25,305	5,546	11,997	11,015	6.62	0.77	0.87
GP-3	28,892	5,778	9,937	9,349	7.10	0.82	0.90
GP-4	30,351	5,255	9,415	8,709	6.84	0.80	0.91
GP-5	28,207	3,599	4,586	4,275	6.96	0.85	0.96
GP-6	28,588	5,335	8,881	7,960	7.24	0.84	0.91
GP-7	28,506	6,158	9,855	9,230	7.58	0.87	0.90
GP-8	26,182	5,805	13,864	12,889	6.87	0.79	0.86
GP-9	31,911	3,405	9,005	8,442	5.05	0.62	0.93
GP-10	16,983	3,897	10,502	9,693	6.42	0.78	0.85

^a^*Richness and diversity were determined based on 0.03 distance.^*b*^Evenness was calculated by dividing Shannon index by Ln(OTUs).*

Groundwater at different depths of the study site was sampled in parallel with the sediment core sampling with a drive-point piezometer system called Retract-A-Tip (AMS, Inc.; Charette and Allen, 2006). The stainless-steel piezometer was driven to the depth of interest, and groundwater samples were pumped through Teflon tubing using a peristaltic pump. Samples for nutrients were collected into 30 mL acid-cleaned scintillation vials after passage through a Pall Aquaprep 0.22 μm capsule filter and stored frozen until analysis. Water properties including temperature, salinity, pH, dissolved oxygen, and Oxidation-Reduction Potential (ORP) were recorded using an YSI 600XLM multiprobe and 650MDS handheld computer. Iron (Fe) was measured using the calorimetric Ferrozine method (Viollier et al., 2000) which fixes Fe in the sample for measurement of Fe^2+^ prior to chemical oxidation for measurement of Fe^3+^ to determine total iron. Dissolved N_2_O concentration was measured by collecting 30 mL of groundwater in 50 mL serum bottles with a pellet of KOH. After vigorous shaking to equilibrate dissolved gases, 10 mL of headspace gas was sampled and injected to a gas chromatograph equipped with electron capture detector (Shimadzu, Kyoto, Japan).

### DNA Extraction, PCR Amplification, and Sequencing

DNA was extracted from 0.5 g of sediment (wet weight) using a Power soil^®^DNA isolation Kit (Mo-Bio Laboratories, Inc., Carlsbad, CA, United States) according to the manufacturer’s instructions. Thermo Savant Fast Prep FP 120 Cell Disrupter (Qbiogene Inc., Carlsbad, CA, United States) was used for cell disruption. PCR was conducted to amplify the V_4_ hypervariable regions of 16S rRNA genes of bacteria and archaea using forward 515F and reverse 806R primers (Caporaso et al., 2011). The reverse primer was modified with 8 bp barcodes, and both forward and reverse primers were added with adapter primers corresponding to the sequencing protocol of the Ion Torrent Personal Genome Machine (PGM). Aliquots of a 25 μL PCR reaction included 12.5 μL 2 × Go-Taq master mix, 1 μL of each primer (5 μM), 1 μL template DNA and 9.5 μL nuclease-free water. The PCR reactions were performed in duplicate under the following conditions: initial denaturation at 95°C for 2 min, 25 cycles of 95°C for 30 s, 55°C for 30 s, 72°C for 30 s, and then a final extension at 72°C for 7 min. A negative control without DNA template was included in each PCR reaction to detect for any contamination of genomic DNA. The amplicons of each sample were pooled and purified with the Wizard^®^ SV Gel and PCR Clean-Up System.

The purified PCR products were quantified using a 2200 Tape Station instrument and D1K reagents (Agilent Technologies, Santa Clara, CA, United States) following the manufacturer’s instructions. Sequencing was performed using an Ion Torrent PGM sequencer with the pooled samples on a 316 chip, following the manufacturer instructions (Life Technologies, Grand Island, NY, United States). The sequence reads with more than one ambiguous nucleotide (N) and incomplete sequences of barcode and 16S primers were removed from a subsequent bioinformatic analysis. The filtered sequences were assigned to each sample based on the barcode.

### Sequence Analyses and OTU Clustering

All sequence reads with complete match of the barcodes and a single mismatch to the 16S primers were retained and then trimmed by removing the sequencing adaptor, barcodes and 16S primer sequences to obtain valid raw reads. The reads were further screened by using the following thresholds: (i) minimum average quality score of 25; (ii) minimum read length of 200 bp; (iii) sequences containing no ambiguous bases; and (iv) maximum homopolymers of 8 bp. Quality-controlled sequences were analyzed in MOTHUR (Version 1.35.1) following standard operating procedures^2^ (Schloss et al., 2009). The Greengene database (gg-13-5-99.align) was used to align and classify the reads. The OTUs containing more than 1% of total sequences were defined as dominant OTUs. Phylogenetic analysis of the dominant OTUs (Top 50 OTUs) was conducted with the software MEGA7.0 and Evolview (He et al., 2016). A heat map was constructed in accordance with the abundance of dominant OTUs using Microsoft Excel.

### Metabolic Inference and Functional Gene Prediction

Phylogenetic Investigation of Communities by Reconstruction of Unobserved States (PICRUSt)^3^, a predictive exploratory tool, combined with the Kyoto Encyclopedia of Genes and Genomes (KEGG) ortholog classification, was used to infer the metabolisms of sediment communities and to generate predicted metagenomes based on the classified 16S rRNA reads of each sample (Langille et al., 2013; Kanehisa et al., 2014). The functional genes in methane, nitrogen and sulfur metabolisms were selected to compare potential functions of microbial communities in the sediments of GP-STE.

### Quantitative PCR (q-PCR) Assay

The q-PCR assays of the 16S rRNA gene were performed to quantify the abundance of bacteria and archaea in the sediment communities. The q-PCR reactions were performed in triplicate within a volume of 20 μL containing SYBR green using Go-Taq qPCR Master Mix (Promega Corporation, Madison, WI, United States) with the primer set of 515-F and 806-R using ABI Prism 7500 Real Time PCR System (Applied Biosystems, Carlsbad, CA, United States). Each reaction was performed in a 25 μL volume containing 2 μL of DNA template, 0.2 μL BSA (0.1%), 1 μl of each primer (20 μM, 515F, and 806R), 12.5 μL of Power SYBR Green PCR Master Mix and 9 μL of nuclease free water (Applied Biosystems, Foster City, CA, United States). The q-PCR cycle was as following: 10 min at 95°C, followed by total of 35 cycles of 95^o^C for 30 s, 55^o^C for 30 s, 72^o^C for 30 s. A standard plasmid carrying the 16S rRNA gene was generated by amplifying 16S rRNA gene from DNA extracted from GPMD sediment and cloned into the pMD-T-18 Vector (Takara, Japan). The plasmid DNA concentration was determined on a Tape Station 2200 (Agilent) and the copy numbers of target genes were calculated directly from the concentration of the extracted plasmid DNA. Ten-fold serial dilutions of a known copy number of the plasmid DNA were subjected to q-PCR assay in triplicate to generate an external standard curve.

### Statistical Analysis

Chao and Shannon estimators were generated in Mothur as proxies of the alpha diversity index. Redundancy analysis (RDA) was performed to examine covariance among environmental variables and the dominant OTUs using the Canoco 5.0 software. Principal coordinate analysis (PCoA) was performed in Mothur software on relative sequence abundance data at the OTU level (Schloss et al., 2009). The vertical variation in environmental parameters and microbial abundance were analyzed using one-way analysis of variance (ANOVA), and significance level was set at α < 0.05.

### Nucleotide Sequence Accession Numbers

The reads of the 16S rRNA genes were deposited in the ENA Short Read Archive under the submission number SRP154289.

## Results

### Environmental Characteristics and Microbial Abundance of the Sampling Site

Depth profiles of environmental characteristics [salinity, temperature, dissolved oxygen (DO), pH, N_2_O, Fe (II), Fe (III), Fe (total), and ORP] and microbial abundance were shown in Figure 1. We found sharp gradients of salinity, DO, and ORP in the groundwater sampled from different depths of the sampling site in the GP-STE. The salinity increased slightly from 17.80 psu at 30 cm to 18.20 psu at 40 cm, and then declined sharply to 1.26 psu at depth of 90 cm. The spatial distribution of salinity indicated that seawater can be transported to the STE via two different ways: vertical permeation from the surface, and horizontal transportation from seawater terminal. The DO profile was measured using an oxygen probe, revealing a sharply decline from 7.3 ppm at 30 cm to 0.14 ppm at a depth of 90 cm. The temperature gradually increased along the profile from 12.70 to 17.00°C. pH value declined sharply from 7.20 at 30 cm to 6.60 at 60 cm, and then increased from 6.60 to 7.20 at 80 cm. The N_2_O concentration varied from 0.06 to 0.13 ppm and peaked at the depth of 60 cm. In addition, the N_2_O concentration was under detection level below depth of 60 cm, Similar to the profile of DO, the ORP value decreased with sediment depth from 157.40 to -127.00 mv. The dissolved Fe peak occurred at the depth of 70 cm where the sand layer was highly enriched with iron oxides. The profile of microbial abundance showed no significant variation (*P* > 0.05) in sediment core [ranging from (0.23 ± 0.03) × 10^9^ to (4.62 ± 0.08) × 10^9^ copies g^-1^]. There was a remarkably high microbial abundance in the aerobic-anaerobic transition zone (AATZ, depth of 50–60 cm) than other depth sediments. The microbial abundance was lowest in the anaerobic zone (GP 8–10, depth of 80–100 cm).

### Similarity-Based Estimation of Archaeal and Bacterial Richness

**Figure 2:**
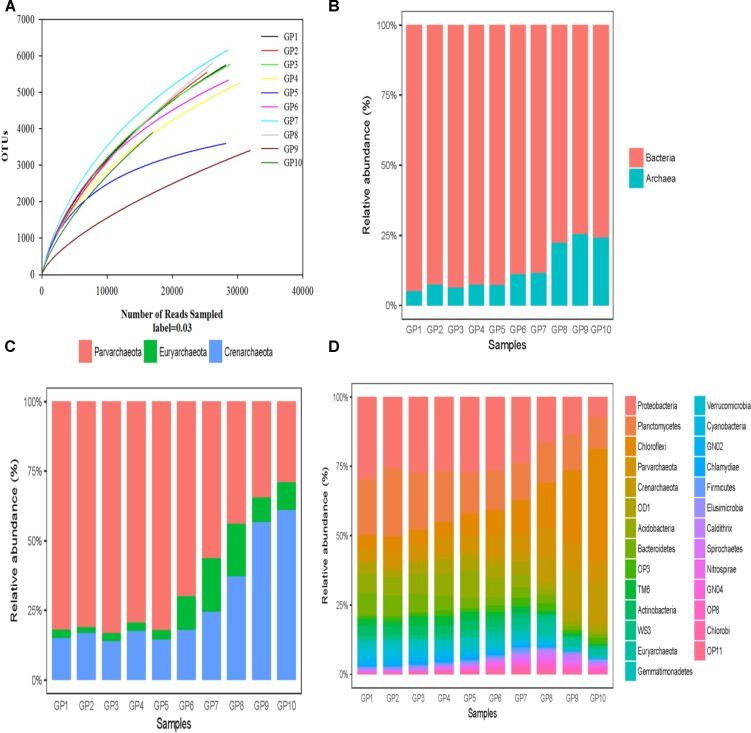
Comparison of bacterial and archaeal communities in the sediments of Gloucester beach. **(A)** Rarefaction curve of 16S rRNA gene analysis with high-throughput sequencing based on OTUs at a 97% sequence identity cut-off. **(B)** Relative abundance of archaea and bacteria based on the numbers of similarity-based OTUs detected in the sediment cores-associated microbial communities. **(C)** Taxonomic classifications of archaeal reads retrieved from sediment cores at phylum level. **(D)** Taxonomic classifications of bacterial reads retrieved from sediment cores at phylum level.

The Ion Torrent PGM generated about 350,000 raw reads of 16S rRNA genes from 10 sediment samples. After removing of noise and low-quality reads, more than 270,000 reads were used for further analysis (the high quality reads in each sample were listed in Table 1). In total, 31,146 OTUs were assigned at a 97% sequences similarity threshold with the most abundant OTUs (>100 reads per OTU) accounting for 49% of all sequences. There were 30,263 singletons accounting for 52.9% of the total OTUs that might have resulted in the high microbial diversity. The number of OTUs ranged from 3,405 to 6,158 across all samples, with sample GP7 (at depth of 70 cm) sediments harboring significantly higher numbers of OTUs than other samples in the sediment core. The Shannon diversity index was also significantly higher in GP7 sediment community than others (*P* = 0.004). The coverage of each sample ranged from 86.0 to 96.0%, with an average value of 89.8%, indicating that the sampling efforts of all samples were sufficient to represent indigenous species. Rarefaction curves showed that the high-throughput sequencing provided sufficient bioinformation to investigate the community composition and diversity of bacteria and archaea in this study (Figure 2A). Similarly, ACE and Chao1 estimators showed clear differences in species richness across the sampling depths, which were about twofold higher than the OTU numbers (Table 1), suggesting that twice as many OTUs may exist in the sediments.

### Taxonomic Classification at the Phylum Level

At a confidence threshold of 80%, 273,103 out of 273,789 qualified reads (count for 99.7%) could be assigned to a known phylum using the Mothur classification program, of which 59,128 belonged to archaea and 213,975 were bacteria. Relative abundance of bacteria decreased from 88.2 to 74.6%, while archaeal abundance increased correspondingly and ranged from 11.8 to 25.4% as the depth increased (Figure 2B). Four archaeal phyla, Crenarchaeota, Euryarchaeota, Parvarchaeota, and an unclassified phylum, were found in the sediment samples with varying relative abundance at different depths. Crenarchaeota was the dominant species in the archaeal communities, constituting up to 60% of the archaeal reads, followed by Euryarchaeota (30%), which were retrieved mainly from bottom sediments (80–100 cm). Parvarchaeota was a minor group, and the relative abundances in the upper samples were higher than those in the lower samples (Figure 2C).

In total, 70 bacterial phyla were recovered from our 9 samples (Supplementary Table S1). When comparing the bacterial communities in different phyla, Proteobacteria (30.70%), Chloroflexi (30.01%), Planctomycetes (8.93%), Bacteroidetes (3.12%), Acidobacteria (1.97%), Cyanobacteria (2.20%), OD1(1.85%) and an unclassified phylum (6.63%), altogether constitute up to 86.6% of the reads affiliated with bacteria (Figure 2D). However, reads belonging to Gemmatimonadetes, WS3, Chlorobi, Nitrospirae, Chlamydiae, Verrucomicrobia, TM6, OP8, Caldithrix, OP3, Spirochaetes, Firmicutes, GN02, and GN04 were found to be the minor groups. The heterogeneous distributions of some phyla along the depth profile in the sediment core were observed. For example, Euryarchaeota, Chloroflexi, and WS3 displayed an increasing trend with depth. In contrast, Proteobacteria, Bacteroidetes, Cyanobacteria and Acidobacteria showed a decreasing trend with depth.

### OTU-Level Community Composition and the Effect of Environmental Factors on the Distribution of Microbial Communities

Based on the OTU analysis using PCoA calculations, both bacterial and archaeal community structures displayed variations across the depth profile of the sediment core (Figure 2). The microbial communities at the top to 30 cm (GP1, GP2, and GP3) tightly clustered together and were different from those below 30 cm depth.

**Figure 3:**
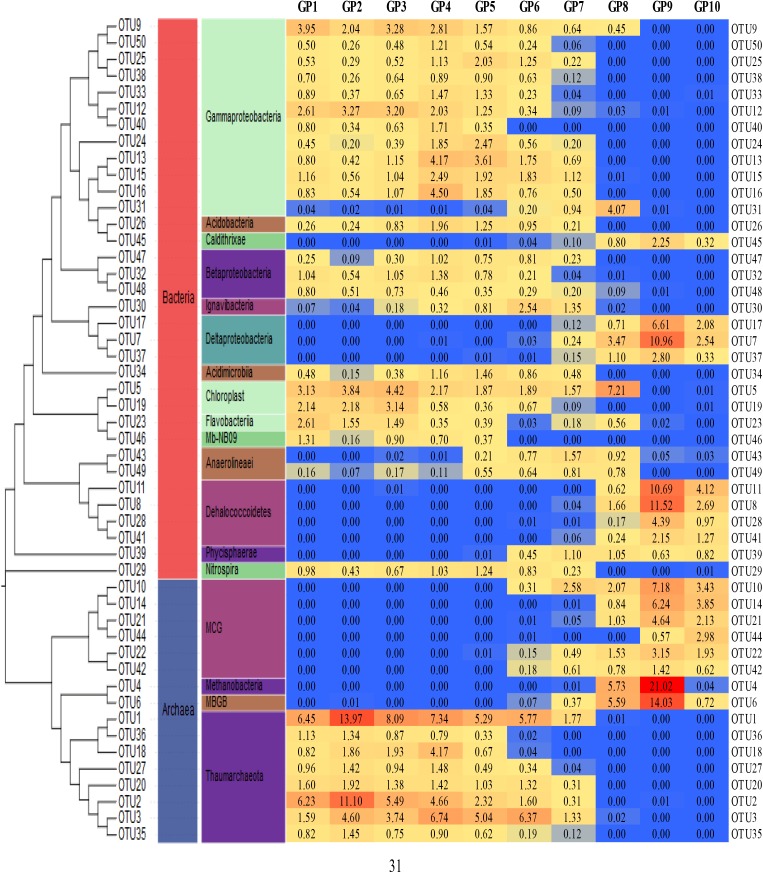
A neighbor-joining phylogenetic tree and heat map of the most abundant 50 OTUs of bacteria and archaea in sediment communities. The OTUs were determined based 97% sequence similarity cutoff.

Figure 3 shows the phylogenic tree of 50 dominant OTUs and their distribution characteristic across all samples. Among the bacteria OTUs, Gammaproteobacteria (12 OTUs), Acidibacteria (OTU26), Betaproteobacteria (OTU47, OTU32, OTU48), Acidimicrobiia (OTU34), Chloroplast (OTU19 and OTU15), Flavobacteriia (OTU23), Mb-NB09 (OTU46), and Nitrospira (OTU29) were dominant in the top to 70 cm sediment layers. In contrast, Deltaproteobacteria (OTU17, OTU7, and OTU37), Dehalococciodetes (OTU17, OTU7, OTU37, OTU41, and OTU39) and Phycisphaerae (OTU39) were dominant members in the 80–90 cm depth sediment layers. Among the archaeal OTUs, Thaumarchaeota (8 OTUs) was mainly distributed in the top to 70 cm sediment layers while MCG Methannobacteria (OTU4) and MBGB (OTU6) were only found in the 80–90 cm depth sediment layers. Moreover, there was a higher variety of dominant OTUs in 49–70 cm depth of sediment layers (GP 6–8) where all of the 50 dominant OTUs were detected. The relative higher diversity in the zone was mirrored by the rarefaction analysis in Figure 2A.

**Figure 4:**
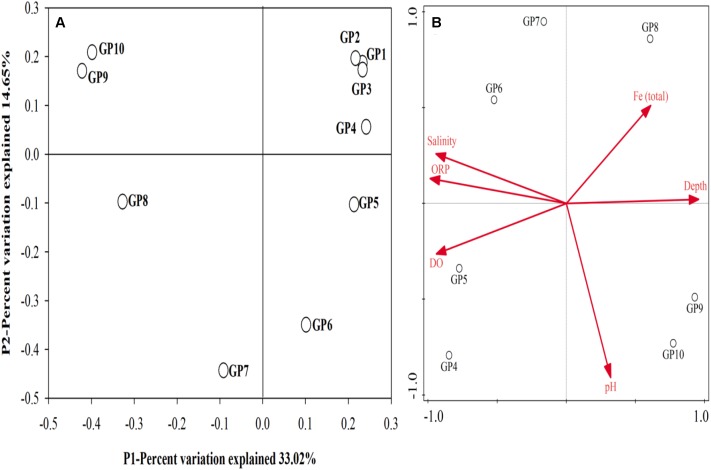
**(A)** Unweighted UniFrac PCoA analyses of the sediment communities in the Gloucester beach based on 16S rRNA gene sequences. **(B)** RDA ordination plot deciphering the relationship between samples and environmental variables in the sediment communities of Gloucester beach. Each symbol represents an individual sample and arrows represent statistically significant environment variables that explain the observed patterns (*P* < 0.05).

Obvious vertical distribution of community composition was revealed by unweighted unifrac PCoA analysis (Figure 4A). PCoA results indicated that bottom and anoxic community assemblages (GP 8–10) were separated from others. Redundancy analysis (RDA) was used to evaluate the relationship between the community composition and the physicochemical characteristics of sediments (Figure 4B). Results showed that sampling depth, DO, ORP and salinity were key physicochemical characteristics affecting the distribution of bacteria and archaea in sediments in the GP-STE.

### Predicted Microbial Functions and Distribution

The metabolic functions based on the classified OTUs were inferred using the PICRUSt program, which categorizes functional gene families in nitrogen, methane, and sulfur metabolism (Table 2). The annotations were classified into 330 functional KEGG or orthology pathways at level 3 (Supplementary Table S2), which included any kind of biological reaction and regulation of gene expression.

**Table 2:** Variation of predicted functional genes in methane, nitrogen, and sulfur metabolisms of the sediment communities along the redox gradients of GP-STE.

	GP1	GP2	GP3	GP4	GP5	GP6	GP7	GP8	GP9	GP10
**Nitrogen Metabolism**					
Ammonia Oxidation-*amoA*	6	4	2	10	0	16	8	2	0	0
Denitrification-*nirK*	1,498	847	1,186	1,231	1,177	1,011	779	253	195	70
Denitrification-*nosZ*	1,394	949	1,249	1,333	1,519	1,784	2,208	2,819	8,331	4,612
DNRA-*nrfA*	1,712	1,125	1,467	1,664	1,941	2,300	2,272	1,386	1,437	788
Nitrogen Fixation-*nifH*	1,833	1,250	1,689	2,212	2,675	2,909	2,714	2,868	4,783	1,257
**Methane Metabolism**					
Methane oxidation-*mmoA*	2,154	1,348	2,065	2,994	2,943	2,038	1,193	418	8	2
Methanegen-*mcrA*	0	0	0	0	0	12	16	439	1,702	84
**Sulfur Metabolism**					
Sulfite oxidation	34	56	53	46	55	47	14	2	0	0
Sulfite reduction-*dsrA*	724	374	575	816	933	1,119	1,277	1,327	1,721	515

*The numbers represent the raw reads of specific predicted function genes and metabolism.*

The genes for aerobic metabolism, including those encoding ammonia monooxygenase (*amoA* genes), methane monooxygenase (*mmoA* gene), and sulfite oxidase, were predicted to have the highest abundance in the oxic surface to 70 cm depth, but the lowest in the deeper depths below 70 cm (GP 8–10). In contrast, predicted abundance of nitrous oxide reductase (*nos*Z) genes, nitrogen fixation (*nifH*) genes, methanegen (*mcrA*) genes, and sulfite reduction (*dsrA*) genes showed an opposite trend to the aerobic metabolism genes. Results also predicted that abundance of *nirK* and *nrfA* gene was higher in AATZ (50–60 cm).

## Discussion

### Environmental Factors Driving Vertical Stratification of Structure and Function of Microbial Communities in the Permeable Intertidal Sediment

In this study, the microbial community compositions of sediments at different depths from surface to 100 cm of permeable sediments were examined using high-throughput sequencing of the 16S rRNA gene. Our results revealed a distinct pattern of vertical distribution consisting of aerobic, aerobic-anaerobic transition (AATZ) and anaerobic zones. The composition of the microbial community in different zone was distinctly different. The high indexes of OTU and Shannon diversity (Table 1) in sediment samples confirmed that the permeable sediments harbor higher microbial diversity similar to estuarine sediments (Liu et al., 2014a,b). Moreover, apparent depth-related differences in measurement of diversity were found in the sediment core, which contrasts with previous studies in estuarine sediments. Furthermore, the microbial diversity index appeared to be significantly higher in the AATZ than in the shallow and deep layers. The variation along the depth profile in the sediment core with seen with PCoA analysis suggests that permeable sediments undergo predictable changes from the surface to bottom layers, which may be significantly different from the distribution of the microbial community structure in less permeable sediments. In the sediment core, bacteria were numerically dominant relative to archaea, with 2–3 orders of magnitude, while the relative abundance of archaea gradually increases from 4.4 to 22% with increasing depth (Figure 2B), suggesting archaea may play more important roles for the biogeochemical processes in the deeper zone than in the upper zone. Moreover, Crenarchaea accounted for more than 50% of the archaeal reads, suggesting that Crenarchaea may play more important roles than other archaeal groups. A similar distribution was also observed in the estuarine sediments (Liu et al., 2014a,b) and mangrove sediments (Bouchez et al., 2013).

It has been suggested that most microorganisms are restricted to specific niches, mainly due to different environmental forces (Lee et al., 2011; Qian et al., 2011; Nelson et al., 2016; Zhang et al., 2017). Our results provide plausible explanations for vertical variations of microbial communities in the permeable sediment. The temporal shift in microbial composition appeared to be associated with the vertical stratification of physiochemical parameters, including temperature, salinity, DO, corresponding significantly to changes in the microbial communities. Previous studies have shown that DO was one of the most important factors affecting the distribution of microbial communities in estuarine waters (Kan et al., 2006), marine subsurface sediments (Durbin and Teske, 2012) and in flooded Paddy Soil (Ludemann et al., 2000). Results of the present study also highlighted the importance of DO in governing the distribution of both bacterial and archaeal communities in the sandy permeable sediments. The DO profile exhibited a sharp decline from 7.3 ppm at 30 cm to 0.14 ppm at a depth of 90 cm and the microbial community distribution was closely related to the DO profile. Above 50 cm, microorganisms were mainly aerobic and facultative aerobic groups, including Gammaproteobacteria, Beltaproteobacteria, Acidobacteria, Chloroplast, Flavobacteria, Thaumarchaeata, and Mb-NB309. At a depth of AATZ (50–60 cm), anaerobic groups appeared gradually increased, showing a co-existence with facultative groups in the transition zone with the highest diversity. Below 70 cm depth, anaerobic microbial groups were dominant in the sediments, including methanogenic archaea, sulfate reducing bacteria, and reductive dehalogenating bacteria.

Previous studies have suggested that salinity is an important factor in shaping the compositions of bacteria communities in estuaries (Crump et al., 1999; Bouvier and del Giorgio, 2002). However, salinity varied with many environmental parameters across the sediment core, including DO, pH, and temperature; the role of salinity has not been obviously discriminated for the distribution of microbial community structures. Furthermore, the salinity in the sandy sediments is in a state of fluctuation due to the influence of tides (Moore, 1999; Robinson et al., 2007). Therefore, the microbial community in the sediments should be periodically impacted by different salinity. The effects of increased salinity on N-cycling microbes and their associated geochemical functions have been previously reviewed (Santoro, 2010). Salinity appeared to select for a less diverse microbial community in most cases. The activities of different functionally microbial groups have different responses to the change of salinity, for example, the rate of DNRA increased when the salinity increased to 10 psu, but the denitrification process was depressed with the same treatment (Laverman et al., 2007). Although our data showed that the salinity was an important factor that influenced the distribution of microbial groups, no clear pattern was observed for how defined groups of bacterial or archaea community shift with the salinity variation. Further study of the salinity effect of on composition and function of microbial communities will increase our understanding of microbial processes in STEs.

### Distribution of Metabolic Potentials Across Redox Gradients

The surface of the core is rich in oxygen, which provides conditions for the aerobic metabolism of microorganisms. The predicted metagenome analysis showed microbial aerobic metabolism, including methane oxidization (Bender and Conrad, 1994), ammonia oxidization (Ward and Jensen, 2014), and sulfite oxidation (D’Errico et al., 2006), mainly occurring in the aerobic zone. Similar to other STE locations (Charette and Sholkovitz, 2002; Roy et al., 2013), a bright red-orange layer of precipitated iron oxide was visible in the sand cores from 55 to 70 cm. The intensity of this oxide layer and the levels of dissolved Fe appeared to fluctuate in both time and depth, which we believe was linked to hydrologic controls, similar to other locations (Michael et al., 2005; Gonneea et al., 2013). In the AATZ, almost all dominantly predicted genes related to denitrification, DNRA and sulfite reduction were observed with relatively high abundance, suggesting both aerobic and anaerobic metabolism could occur in the transition layer. Therefore, the aerobic-anaerobic interface should be a hotspot for microbial functional metabolism. Microbial anaerobic metabolic pathways were primarily found in the anaerobic zone, for example sulfate reduction and metagenesis. Overall, the predicted microbial functions also presented a typical stratification feature, and function prediction showed that the variation of potential microbial metabolic function was matched to the community shift in the sediment core, showing a unification of the distribution of microbial diversity and functional metabolism.

Oxygen plays an important role in regulating the function of microorganisms. In fact, most of the microbes were of the facultative respiration type, capable of using a variety of electron acceptors (Hogg, 2005). Under low oxygen conditions, a variety of electron acceptors can be used alternately to improve the efficiency of metabolic productivity (Jose, 2004; Canfield et al., 2005). In particular, the concentration of dissolved oxygen will fluctuate slightly, especially in the STEs where fresh ground water and sea water are mixed (Moore, 1999; Gonneea and Charette, 2014). Under these conditions, the metabolism of microorganisms will also be significantly regulated by dissolved oxygen in the pore water. Many microorganisms can alternate or simultaneously carry out aerobic and anaerobic respiration under low oxygen conditions. The higher diversity of both OTUs and predicted functions demonstrated that aerobic and anaerobic metabolism existed concurrently in the AATZ. Combined with the diversity and the predicted functions of the microbial communities, we propose that the aerobic-anaerobic interface in the highly permeable of sandy sediments is a hotspot of microbial geochemical reactions, regulating nutrient discharges to surface water.

## Author Contributions

YH and BS performed the research. YH, JW, and SW analyzed the data. YH and BS wrote the manuscript and all co-authors substantially contributed to commenting and revising it. All authors read and approved the final manuscript.

## Conflict of Interest Statement

The authors declare that the research was conducted in the absence of any commercial or financial relationships that could be construed as a potential conflict of interest.

## Footnotes

1 http://picrust.github.io/picrust

2 https://www.mothur.org/wiki/Download_mothur

3 http://picrust.github.io/picrust
