# Supplementary material for: Vertical Stratification of Sediment Microbial Communities Along Geochemical Gradients of a Subterranean Estuary Located at the Gloucester Beach of Virginia, United States
Source: Front Microbiol. 2019 Jan 11;9:3343. doi: 10.3389/fmicb.2018.03343 (PMC6336712; doi:10.3389/fmicb.2018.03343)
Supplement: Supplementary file 1 [file Data_Sheet_1.docx]

**Supplementary material**

**Frontiers in Microbiology**

**Environmental gradient shaped the vertical stratification of microbial community structure and potential function in** **permeable intertidal sediments, Gloucester Point**

Yiguo Hong^1^*, Jiapeng Wu^1^, Stephanie Wilson^2^ and Bongkeun Song^2^*

*^1^College of Environmental Science & Engineering, Guangzhou University, Guangzhou, 510006, P.R. China.*

*^2^Department of Biological Sciences, Virginia Institute of Marine Science College of William & Mary, Gloucester Point USA*


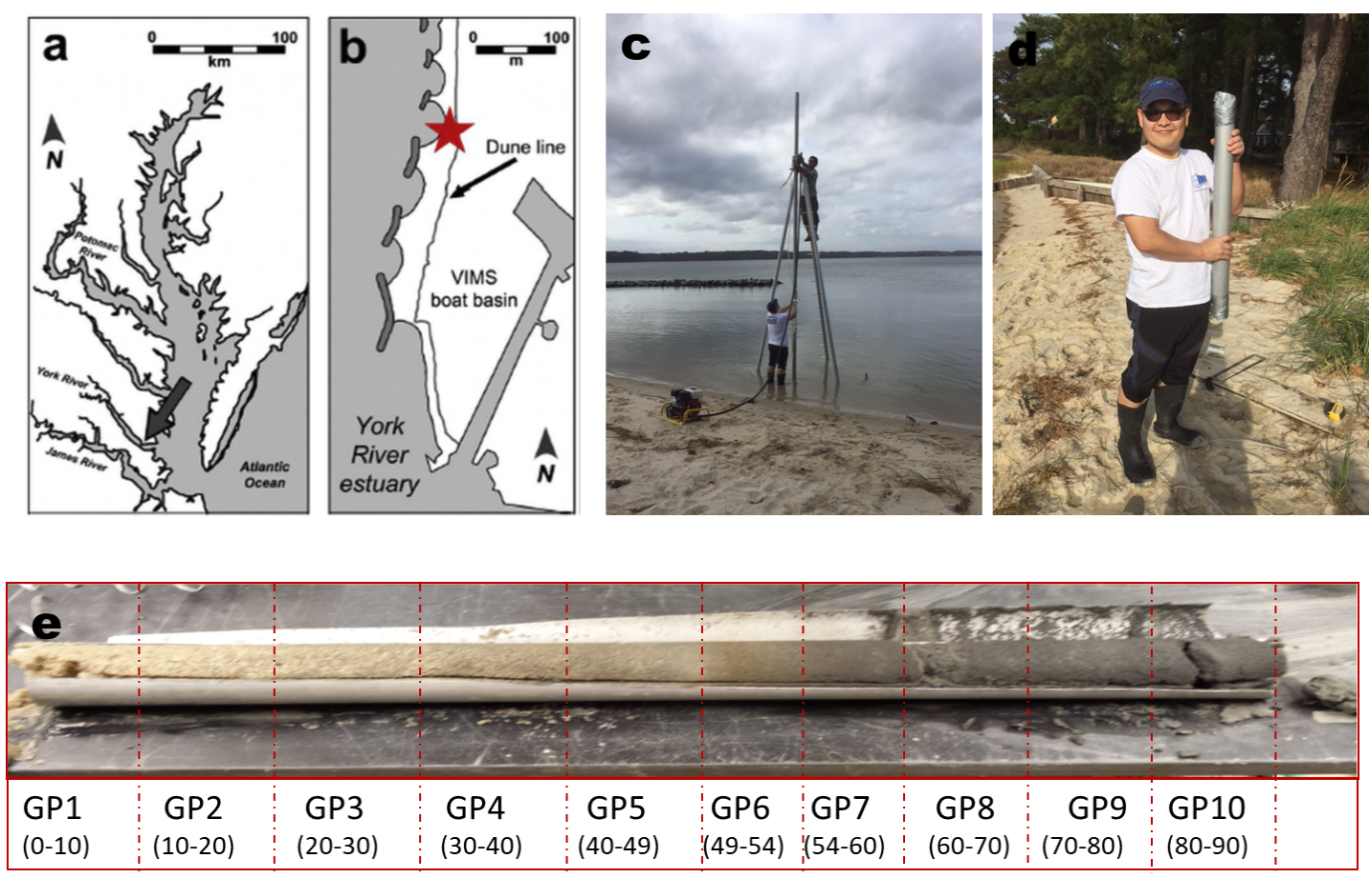


Fig.S1 The Gloucester Point site on the York River estuary (which drains to the Chesapeake Bay). (b) The study site is on VIMS beach. Sediment core were taken at the mid-tide line in the location indicated by the star. (c) and (d) are the scenes of working for sediment core collection. (d). Sediment core was fractioned into 10 subsamples.
